# ‘Suffering for the sins of others’: Lucius D. Bulkley, *Syphilis Insontium*, and disease destigmatisation in the progressive era United States

**DOI:** 10.1017/mdh.2024.44

**Published:** 2025-01

**Authors:** Elliott Bowen

**Affiliations:** Independent Scholar

**Keywords:** Syphilis, Sexually transmitted infections, Stigma, Destigmatisation, Public health, United States

## Abstract

Historical research on efforts to reduce the stigma associated with venereal disease (VD) generally dates these campaigns back to the 1930s. Within the United States, one of the earliest attempts to detach VD from its traditional association with sexual immorality occurred during the late nineteenth- and early twentieth-century, when the New York City dermatologist Lucius Bulkley coined the term *syphilis insontium* (‘syphilis of the innocent’) in the hopes of demonstrating that many of those who contracted this disease did so through non-sexual contact. Gaining widespread acceptance within the medical community, Bulkley’s ideas served as the intellectual foundation for a discursive assault on the prevailing belief that syphilis constituted the ‘wages of sin’—one designed to destigmatise the disease and to promote more scientific responses to it. However, the effects of this anti-stigma rhetoric were often counterproductive. Encouraging doctors to discern ‘innocence’ or ‘guilt’ through assessments of a patient’s character, *syphilis insontium* often amplified the disease’s association with immorality. With the passage of time, physicians became increasingly aware of these problems, and in the 1910s, a backlash against Bulkley’s ideas emerged within the American medical community. Yet even with the resultant demise of his destigmatisation campaign, discourses of ‘innocent syphilis’ continued to circulate, casting a long shadow over subsequent stigma reduction efforts.

On 5 June 1900, a New York City dermatologist named Lucius Duncan Bulkley presented a paper at the annual meeting of the American Medical Association in Atlantic City, New Jersey titled ‘Syphilis as a Non-Venereal Disease’. In his opening remarks, Bulkley praised the country’s recent progress against infectious disease, noting that as a result of the creation of public health boards and the passage of contagious disease laws, ‘we no longer have the wholesale sweep of epidemics which occurred before’.^1^ Despite this progress, however, there was one disease that remained outside the purview of health authorities and that was ‘unchecked by sanitary control’: syphilis.^2^ Though it ‘counted its victims by tens and hundreds of thousands’, syphilis was being ‘ignored’, and had ‘been allowed to pursue its unbridled course’, without any official intervention.^3^

In explaining the source of this public inaction, Bulkley drew attention to ‘the shame which has too often checked discussions of the subject’.^4^ The belief that syphilitic sufferers were ‘guilty of sexual transgression’, he argued, had ‘hampered’ efforts to reduce its spread.^5^ Yet this need not be the case, for as doctors were increasingly discovering, syphilis could be spread by means ‘other than unlawful venereal acts’.^6^ As evidence of how it could be ‘innocently given to individuals’, Bulkley recounted cases in which breast-feeding, kissing, blood-letting, vaccination, tattooing, circumcision, glass-blowing, and the use of infected household utensils had resulted in the transmission of syphilis.^7^ Because the disease could easily be acquired ‘quite apart from any sexual relations’, Bulkley concluded that ‘there need be no stigma connected with the discussion of it’.^8^ Indeed, given the ‘mass of facts’ testifying to the possibilities of ‘innocent infection’, it was simply ‘too late in the history of science and of humanity to stigmatise the disease as “venereal”, and on that account withhold scientific protection from thousands of innocent sufferers’.^9^

Bulkley was not alone in this belief. Those attending his presentation agreed that syphilis was frequently ‘non-venereal’, and that by ‘dropping the sexual view of it’, doctors might help overcome the ‘odium’ attached to the disease.^10^ Beyond this, however, there was little consensus. While some argued that making syphilis a reportable malady would reduce the stigma associated with it, others believed that this would have the opposite effect. Requiring doctors to provide public health authorities with information about the syphilitic men and women under their care, one respondent predicted, would actually ‘stigmatize these patients’ in a way that discouraged them from seeking medical aid. Seconding this opinion, another discussant observed that because ‘the disease is a stigma’, most doctors would be reluctant to report private cases of syphilis anyway.^11^

Exchanges such as these were fairly common in late nineteenth- and early twentieth-century medical circles. Though historians generally date the earliest venereal disease (VD) destigmatisation efforts to the 1920s and 1930s, the decades prior to this were in fact replete with pleas for greater social acceptance of men and women with syphilis.^12^ Between the 1890s and 1910s, physicians seeking to combat the view that this disease constituted the ‘wages of sin’ often invoked the concept of ‘innocent infection’, which Bulkley popularised with the publication of his 1894 book *Syphilis Insontium* (*Syphilis of the Innocent).* Though widely referenced in historical scholarship, this concept’s career has been insufficiently mined, leading to inconclusive and at times conflicting assessments of its impact. Somewhat paradoxically, *syphilis insontium* has been both lauded for making treatment ‘more respectable’ and denigrated for generating ‘new fears’ about the dangers of VD.^13^

How effective was *syphilis insontium* as a destigmatisation strategy? In her influential essays *Metaphor as Illness* and *AIDS and its Metaphors*, Susan Sontag famously argued against such approaches to stigma reduction. ‘Victims suggest innocence’, she declared. ‘And innocence, by the inexorable logic that governs all relational terms, suggests guilt’.^14^ Following Sontag, one critic of *syphilis insontium* has written that ‘the very insistence that some sufferers were “innocent”’ implied that ‘the rest were guilty’.^15^ Along similar lines, Monika Pietrzak-Franger argues that Bulkley’s ideas were ‘imbued with moralist views, which have the distribution of blame at their core and reek of judgmentalism’.^16^ Finally, in a comparison of messages conveyed through early twentieth-century sex education films and contemporary AIDS documentaries, Elisabet Björklund observes that ‘discourses around innocence and victims can be destructive, even when the blame is not explicitly laid on anyone’.^17^

Other research challenges these claims. A recent study of 1930s VD campaigns, for example, finds that discourses of ‘innocent infection’ succeeded in turning attention away from questions of blame and guilt – in some cases, by demonising the bacterium (*Treponema pallidum*) that causes syphilis.^18^ As this finding suggests, in order to determine whether *syphilis insontium* intensified or reduced the stigma attached to syphilis, it is necessary to place this innovation in disease nomenclature within the context of the broader anti-stigma rhetoric that shaped its meaning and use, and to consider the concept’s deployment in specific clinical settings. In keeping with other scholarship that has subjected Sontag’s ideas to empirical scrutiny, in what follows, I aim to understand the formulation and practice of *syphilis insontium* within late nineteenth- and early twentieth-century American medicine.^19^

To challenge the prevailing assumption that syphilis was a penalty for sexual immorality, Bulkley and his allies argued that many syphilitic men and women were ‘suffering for the sins of others’.^20^ Filling the medical literature with cases of ‘innocent infection’, they succeeded in convincing many of their colleagues that people with syphilis were often deserving of ‘our kindest and most charitable friendship and care’.^21^ But Bulkley was unable to dislodge the ‘wages of sin’ mentality. While not always generating discourses of blame, his portrayal of ‘innocent’ syphilitics as victims worked to reinforce syphilis’ existing associations with immorality, thus enabling all manner of hostile behaviour towards those deemed ‘guilty’.^22^ Moreover, as Bulkley’s ideas moved into the clinic, they imbued questions of personal morality with great diagnostic importance, encouraging doctors to discern ‘innocence’ or ‘guilt’ through assessments of a patient’s character and conduct. Unable to escape broader discourses of shame and blame, the efforts of Bulkley and his followers made it difficult to break syphilis’ reputation as the ‘wages of sin’.

In documenting this failed effort, this article proceeds in four parts. It begins with a historical overview of responses to syphilis in the late nineteenth- and early twentieth-century US, highlighting the conflict between moral and medical approaches to disease control. From here, I chronicle the origins and development of Bulkley’s ideas about ‘non-venereal syphilis’ – along with the medical community’s reception of these. As will be seen, Bulkley’s commitment to the concept of *syphilis insontium* reflected a fairly widespread desire to alleviate the feelings of shame and guilt experienced by his syphilitic patients – or at least those found to have been ‘falsely accused’ of sexual misconduct.^23^ Focusing on the clinical application of *syphilis insontium*, the paper’s third section shows that while Bulkley’s ideas often proved beneficial for those patients awarded a diagnosis of *syphilis insontium*, their destigmatising energies were never extended to those found ‘guilty’ of sexual transgressions. As such, syphilis’ pre-existing connection with immoral behaviour remained intact. Only in the 1910s and 1920s, when a strong reaction against Bulkley’s writings emerged, did the medical profession begin to discard the moral criteria used to levy diagnoses of *syphilis insontium.* As the paper’s fourth section shows, this reaction set the stage for a new era in the history of *syphilis insontium* – one involving a much more thoroughgoing critique of the discourses of shame, blame, and guilt that Bulkley and his colleagues had initiated. As will be seen, though Bulkley’s approach to stigma reduction had lost purchase by the 1920s and 1930s, the concept of *syphilis insontium* lived on, taking on new meanings and opening up new possibilities for stigma reduction.

## The ‘venereal peril’ in late nineteenth- and early twentieth-century American society

The late nineteenth century was a time of growing medical and social interest in syphilis. Previously regarded largely as a private matter, in the decades following the US Civil War, doctors began to brand the disease ‘a menace to the national welfare’ and a ‘degenerating force on the human race’.^24^ In part, syphilis’ transformation into a bona fide public health threat reflected changes in medical knowledge. With the establishment of venereology as a medical speciality, doctors uncovered evidence of the relationship between VD and an array of medical conditions (including infertility, insanity, and infant blindness) previously attributed to other causes.^25^ Such discoveries convinced members of the medical profession that syphilis was ‘a much more common disease than is generally thought’, and fuelled a host of rather bombastic claims within the medical press.^26^ Writing from New York City, in 1901, the prominent urologist Abraham Wolbarst warned that

The flower of our land, our young women…are being mutilated and unsexed by surgical life-saving measures because of these diseases, particularly gonorrhea. Young men are filling our institutions for the defective and insane, because of the ravages of syphilis. Sightless children and grown men and women are crying out in their blindness against this arch crime of gonorrhea; the souls of infants born only to die or to suffer, cry out against the infamy of uncured syphilis.^27^

The inescapable conclusion was that VD was ‘alarmingly on the increase’, and that the US was confronting a ‘great Red Plague’.^28^

Fears of a looming VD epidemic persisted through the early twentieth century – an era marked by the discovery of syphilis’ bacterial cause (*T. pallidum*, discovered in 1905), a new serological test for diagnosing the disease (the Wassermann test, invented in 1905), and a new drug for treating it (Salvarsan, a therapy created in 1910).^29^ Importantly, these fears stemmed not just from medical and technological advancements but also from anxieties related to broader economic, demographic, and cultural changes in US society.^30^ When documenting the ‘appalling prevalence’ of syphilis, VD specialists frequently positioned this disease as the cause of many broader phenomena that troubled the country’s elites – including a perceived crisis of family life tied to the declining fertility of native-born white women, rising rates of divorce, and increasing rates of pre- and extra-marital sex.^31^ When combined with developments in industrial capitalism that brought increasing numbers of southern and eastern Europeans to the nation’s urban centres, these trends fuelled a eugenic cry of ‘race suicide’, which many attributed to the ‘evil’ of syphilis.^32^

To stem the tide of America’s ‘venereal peril’, concerned physicians nationwide launched the ‘social hygiene’ movement.^33^ Seeking to end the ‘conspiracy of silence’ surrounding VD, their attempts to stop the spread of syphilis and gonorrhoea took different forms reflecting two distinct approaches to disease control: one rooted in Victorian norms and the belief that syphilis constituted a ‘punishment for sexual immorality’, and a second that reflected a ‘new, secular, scientific paradigm’.^34^ As historical analyses have shown, social hygienists espoused a generally conservative view of society, and in the conflict between ‘sin’ and ‘science’, the former approach generally triumphed. Repressive and highly coercive, responses to syphilis (which included aggressive crackdowns on prostitution, the introduction of sex education into the public school system, the passage of eugenic marriage laws, and immigration restrictions designed to prevent those with syphilis from entering the country) generally targeted marginalised populations, and treated the disease as a condition of the ‘other’.^35^ Though calling for a dispassionate, scientific approach to VD, social hygienists frequently used fear-based approaches to compel adherence to a strict, family-centred Victorian sexual ethic.^36^ Often, they wielded stigmatisation as a public health tool, believing that this could play a constructive role in reducing rates of syphilis.^37^

Despite this, there were efforts to counter the ‘wages of sin’ metaphor. When encountering people with syphilis, those physicians who sought to look at the disease ‘from a purely scientific standpoint’ urged their colleagues to ‘forget the moral deficiencies of [the] patient and think only of his sickness’.^38^ As Allan Brandt notes, hospitals sometimes refused to collect VD statistics so as to ‘spare parents and families from the social stigma attributed to them’.^39^ Similarly, in his analysis of early twentieth-century responses to VD in Scotland, Roger Davidson remarks on how treatment facilities designed to allow for the ‘rapid handling of patients’ reflected ‘a desire to minimize social stigma and maximize patient compliance’.^40^ Outside the realm of clinical medicine, novelists and other writers also sought to overturn syphilis’ dominant representation as a ‘shameful disease’ caused by the ‘sin of unchastity’.^41^ One prominent example of this was Sarah Grand’s *The Heavenly Twins*, a popular novel whose female protagonist experiences a syphilitic infection presented not as the result of ‘sinful choice’, but rather as ‘a product of naïve innocence’.^42^ Advancing a metaphor of blameless, innocent syphilis, Grand’s story urged readers to reevaluate the ‘sin-syphilis connection’ by highlighting its role in maintaining the patriarchal structures of Victorian society.^43^

## ‘In many a case, an otherwise distressing stigma can be lifted’

It was within this environment of heightened interest in syphilis that Bulkey’s destigmatisation campaign unfolded. The problem of disease stigma first came to Bulkley’s attention during his early years as a dermatologist in New York City. After graduating from the College of Physicians and Surgeons of New York in 1869, Bulkley travelled to Paris, Berlin, and Vienna in search of advanced training in dermatology.^44^ Returning to his native New York City in 1874, he quickly took up private practice, along with a position as an attending physician in the Skin Department at Demlit Dispensary. Over the course of the next several years, he regularly encountered patients who had been denied admission to local hospitals on the grounds that eczema, psoriasis, and other skin disorders were ‘objectionable’.^45^ Hoping to bring an end to such discriminatory practices, in 1882, Bulkley helped found the New York Skin and Cancer Hospital.^46^ He remained there until his death in 1928.^47^

The training Bulkley received enabled him to acquire a great deal of clinical knowledge of syphilis. In his first published paper on the subject, he reported on the case of a woman who contracted syphilis ‘after smoking the pipe of a boarder’.^48^ From this emerged a life-long obsession with ‘extra-genital chancre’ – a diagnostic category used to describe cases of syphilis resulting from something other than penile-vaginal intercourse. Between 1880 and 1907, Bulkley published more than a dozen papers on this ‘unusual’ form of infection.^49^ Documenting cases that resulted from ‘bites given during quarrels’, from the use of cigars ‘charged with syphilitic virus’, and ‘by wearing the clothes of others’, he came to see sexual intercourse as only one among many modes by which syphilis could be transmitted.^50^ In a paper published in 1882, Bulkley offered up an early iteration of what would soon come to be the refrain of his entire oeuvre:

Syphilis is no longer to be looked upon as exclusively a venereal affection; multitudes of individuals suffer from it who are wholly innocent of sexual transgression, and in addition to the many who receive it in lawful coitus from those who have been unfaithful, the number of recorded instances of extra genital chancres has multiplied, of late years, beyond the conception of one who has not closely watched the matter. I have myself seen chancres upon the arms, breast, cheek, chin, finger, lip, thigh, tongue and tonsil, and others have observed them on almost every part of the integument.^51^

Syphilis, according to Bulkley, was something that could be acquired ‘as innocently as the child contracts measles, scarlet fever, or whooping cough’.^52^

This was quite a novel interpretation of extra-genital chancre. To be sure, Bulkley was not the first physician to argue that syphilis could be transmitted non-sexually.^53^ But prior to the 1880s, the consensus of professional opinion was, first, that extra-genital infection was incredibly rare, and second, that this could almost always be traced back to ‘unnatural methods of sexual indulgence’.^54^ Though some cautioned that these cases were ‘the result of accident and not of unnatural practices’, the consensus within the American medical community was that extra-genital forms of syphilis were almost always a product of ‘depraved habits’.^55^ This was especially true in the case of anal chancres observed in men, which were universally believed to result from ‘criminal intercourse’.^56^ As awareness of this and other kinds of extra-genital chancre increased in the 1880s, some doctors feared that the United States was becoming a place where ‘unnatural methods of sexual indulgence are sufficiently indulged in’ to such an extent that it would ‘soon rival continental Europe in the number of these cases’.^57^

In contrast to these theories, Bulkley presented a largely desexualised account of extra-genital chancre. To illustrate how common non-venereal syphilis was, in several publications, Bulkley discussed the case of a man purportedly infected through the use of a public bathing suit. In his first encounter with this patient, Bulkley found that despite his obvious syphilitic symptoms, he ‘had no sore on the penis’.^58^ When coupled with the man’s insistence that ‘he had had no venereal exposure in any manner whatever’, the absence of a penile lesion initially left Bulkley quite confused. Further inspection, however, revealed a ‘raw point…about an inch behind the anus’.^59^ Through questioning, Bulkley learned that he had long suffered from itching in the sacral region. Armed with this information, Bulkley then advanced his explanation of the case:

some six weeks before his visit he had been bathing, and, quite contrary to his usual custom, he had worn a strange suit of bathing clothes, and on account of the itching in that region he had rubbed and scratched the part vigorously through the bathing trousers; the sore developed a few weeks after this single wearing of the public bathing suit at Coney Island. It is easy to understand how a previous bather with mucous patches at the anus had left the secretions on the garment, and the exertions of rubbing the dampened cloth upon the fissure readily afforded the best possible opportunity for infection.^60^

From this resulted a ‘pretty severe attack of syphilis’.^61^

Bulkley’s explanation of cases like this stretches the bounds of credibility. As scientists have known since the mid-twentieth century, the microorganism that causes syphilis (*T. pallidum*) is unable to survive outside an animal or human host for more than an hour or two.^62^ Yet while erroneous in the light of modern-day scientific knowledge, Bulkley’s concept of *syphilis insontium* reflected many of the broader trends in medicine and public health during the late nineteenth and early twentieth centuries. With the rise of germ theory in the 1870s and 1880s, medical scientists gained new insights into how mundane bodily habits such as spitting, sneezing, coughing, kissing, and even breathing factored into the spread of infectious maladies as varied as tuberculosis, diphtheria, and smallpox. Informed by these bacteriological discoveries, strategies of epidemic management increasingly focused on *indirect* methods of disease transmission, and with this, fears of ‘healthy carriers’ (such as the infamous ‘Typhoid Mary’) and other undetected spreaders of infection increased dramatically.^63^ Though some spoke out against what they saw as a misguided ‘microbe craze’, the idea that apparently healthy people might harbour deadly bacteria shaped much of the era’s public health activism.^64^

Bulkley’s thinking bore the mark of these concerns. Like other leading hygienists of the day, he believed that the conditions of modernity had produced a world where infectious diseases had gained access to ‘every conceivable circumstance and surrounding of life’.^65^ Although he rejected ‘popular’ ideas about syphilis being transmitted through privy seats, public urinals, and ‘normal secretions’ like saliva, tears, or milk, Bulkley nevertheless believed that ‘the opportunities for its propagation seem to be multiplied’.^66^ In his writings, he divided *syphilis insontium* into three basic subtypes: (1) *syphilis economica*, (2) *syphilis brephotropica*, and (3) *syphilis technica.* The first of these headings encompassed infections contracted within domestic settings, and included kissing – which could spread disease whenever an infected person’s mouth contained mucous patches full of syphilitic ‘poison’.^67^ The second type of infection most often resulted from ‘the custom of tasting the nursing bottle which has been in the mouth of a syphilitic infant’.^68^ *Syphilis technica* was most often spread via the use of infected tools and instruments – including the barber’s razor, the dentist’s forceps, the orchestral musician’s mouthpiece, the glassblower’s pipe, and the surgeon’s knife.^69^ Given all of these ‘innumerable’ modes of transmission, Bulkley wondered how anyone managed to avoid syphilis:

With a poison so freely secreted from mucous lesions, and so virulent, and capable of being transported and introduced in so many different ways, and, as far as is known, endowed with the possibility of being preserved for an indefinite period, the only wonder is that cases of non-venereal communication of syphilis are not even more frequent than they are now known to be.^70^

As Monika Pietrzak-Franger points out, Bulkley’s concept of *syphilis insontium* needs to be understood in the context of his profound discomfort with the conditions of modernity. His ideas, Pietrzak-Franger notes, pathologised all manner of domestic, social, and industrial interactions, transforming urban spaces into a ‘pox-ridden monster’.^71^ Yet in contrast to some of his peers, Bulkley was uninterested in debating questions of prostitution or law. His *raison d’etre* was stigma reduction. Opposed to the idea that the disease was always a ‘just penalty for sexual transgressions’, he positioned *syphilis insontium* as a means of overturning the prevailing ‘wages of sin’ mentality that governed popular and professional attitudes towards the disease.^72^ As a diagnostic concept, this would draw attention to the fact that syphilis was often seen in persons of ‘unblemished purity’, while at the same time helping to lift the ‘crushing weight’ of guilt, shame, and self-loathing from the minds of those syphilitics ‘falsely accused’ of sexual improprieties.^73^ His central hypothesis was that ‘less stigma will be attached to the disease, when it is well recognised that many acquire it innocently’.^74^

Bulkley’s stigma reduction efforts, it should be noted, began well before the concept of destigmatisation was formally articulated in medical or other social settings.^75^ But his writings reflect many of the same impulses and concerns motivating contemporary anti-stigma rhetoric.^76^ By campaigning for a change in venereological nomenclature, Bulkley and his allies sought to *normalise* syphilis – that is, to present the disease as something *anyone could contract.* ^77^ This message was a recurring theme in discussions of *syphilis insontium*; as Bulkey himself wrote, ‘no purity of character or correctness of living insures against the possible acquiring of the disease’.^78^

Normalising syphilis meant publishing articles on the disease in which ‘nothing of a sexual nature is touched upon’.^79^ Stressing ‘the non-sexual aspect of lues’ would encourage both doctors and the broader public to think of syphilis as ‘something more than venereal disease’.^80^ Among other benefits, making others more aware of *syphilis insontium* would enable more accurate and timely diagnosis, as the stigma attached to syphilis was often ‘an obstacle to its recognition where such sexual transgressions could not reasonably be suspected’.^81^ Many of those infected with syphilis would need no longer live ‘ever in fear that the eruption present is caused by their sin’, and diagnoses of *syphilis insontium* would also prevent the ‘withhold[ing of] scientific protection from thousands of innocent sufferers’.^82^ From a public health standpoint, stigma reduction would also open the doors to more open, concerted action against the disease; as one of Bulkley’s colleagues observed, ‘the main difficulty which we have to encounter in doing anything to regulate the disease is the association with venereal disease’.^83^ By removing this association, *syphilis insontium* would save lives by helping assert the ‘rights and claims’ of those sufferers who were ‘wrongly suspected’ of acquiring the disease through sexual intercourse.^84^

This, broadly speaking, is how Bulkley and his followers saw their anti-stigma efforts. To achieve their goals, they urged their colleagues to thoroughly investigate all possible instances of non-venereal syphilis. Though aware that it was often ‘impossible to determine’ whether an infection had a venereal or non-venereal origin, Bulkley believed that ‘with sufficient care and patience this can be done…and in many a case an otherwise distressing stigma can be lifted from an innocent person’.^85^ ‘I believe if we are on the lookout for these cases’, he predicted,

They will be more frequently found, and many otherwise inexplicable cases of syphilitic infection will be cleared up, and some innocent persons relieved of the suspicion of having contracted the disease by venereal acts.^86^

As more and more cases in which the disease’s victims had been ‘wrongly suspected’ of sexual transgressions came to light, the public would come to learn that there was ‘nothing in syphilis…which warrants the stigma which almost invariably attaches itself to the individual affected therewith’.^87^

In addition to insisting that doctors search out all possible non-venereal sources of infection, Bulkley also charged his colleagues with the task of imparting their knowledge of *syphilis insontium* to lay audiences. In order to convince society at large that a diagnosis of syphilis ought not unnecessarily ‘impugn the purity of life of the one afflicted’, the work of destigmatisation would need to extend well beyond the clinic.^88^ Having accumulated ‘facts’ attesting to the realities of non-sexual transmission, medical scientists would then seek to ‘arouse the public, in order that masses may be enlightened and guarded against this “great black plague” of venereal diseases’.^89^ With men of science leading the way, a ‘campaign of education and enlightenment’ could commence, and with this, the masses would no longer be ‘left entirely in the dark’ on the subject of syphilis.^90^ Speaking directly to his colleagues, in a 1900 address, Bulkley argued that doctors had a duty to combat faulty ideas about syphilis:

The sooner you can get the idea abroad that syphilis is frequently acquired otherwise than by sexual intercourse, the sooner will it be recognised that there need be no stigma connected with the discussion of it or its restriction by law.^91^

In spreading information about extra-genital chancres, then, doctors could clear away lay misperceptions and replace these with truth.

The medical world’s response to Bulkley’s writings was immediate and highly favourable. While some accused him of exaggerating both the frequency and importance of non-venereal syphilis, most investigators agreed that this was much more common than previously believed and credited him for changing their understandings of extra-genital chancre.^92^ What followed was an outpouring of medical literature detailing the experiences of ‘entirely innocent’ patients suffering from non-venereal chancres.^93^ Most of these studies not only cited Bulkley’s work but praised him for inaugurating a new era in venereological research.^94^ With astonishing rapidity, a ‘large mass of literature on this subject’ came into existence, and at its core were two bedrock beliefs: 1) extra-genital chancres were more widespread than previously believed and 2) the origins of these were frequently non-sexual.^95^ Distancing themselves from ‘the *old idea* that these chancres are contracted by bestial practices’, doctors amplified Bulkley’s claim that it was both ‘incorrect and unjust’ to assume sexual immorality on the part of those afflicted with extra-genital syphilis.^96^ Articulating the new wisdom, a review article published in 1901 declared as follows:

Extra-genital chancres, which were formerly so rare, are rapidly becoming distressingly common. They are found in locations which preclude the suspicion of unnatural practices; they are observed on the lips and in the throats of irreproachable women; they are not rare in the aged in whom sexual passion is but a memory.^97^

Some went so far as to say that when dealing with extra-genital chancres, ‘it is seldom that we need suspect unnatural intercourse as a cause’.^98^ As one writer put it: ‘it is in only a very small minority of [extra-genital cases] that any suspicion of unnatural sexual practices needs to be entertained’.^99^

Just as was true for Bulkley, what made extra-genital syphilis so appealing to these physicians was its destigmatising potential. Like Bulkley, his followers argued that many of the disease’s ‘unfortunate’ victims were ‘more sinned against than sinning’.^100^ Their hope was that in documenting cases of non-venereal infection, they could help foster more positive, sympathetic attitudes towards people with syphilis. As a doctor from California explained:

If there had been more cases of extra genital infection, society, which at present even tabooes the name, would have looked upon the disease in its proper light, not as a punishment of vice, and of necessity as an indication of loose morality, but as a constitutional disease with the possibility of it being acquired by both a mediate and immediate manner of infection; possessing to its victims a danger, reaching into the lapse of years, and capable of being transmitted to their progeny.^101^

## ‘Many respectable people acquire syphilis innocently’

As their campaign progressed, Bulkley and his allies saw signs of attitudinal improvement everywhere they looked. In 1907, Bulkley declared that it was ‘now [a] well recognised fact that syphilis is not necessarily a venereal disease’, and that as such, its contraction no longer carried with it any ‘stigma, or imputation of immoral or impure conduct’.^102^ Echoing this observation, other doctors reported that syphilis had ‘been shorn of much of its former odium’, and that ‘the views of the majority of medical men have been changed’.^103^

The presumption that many extra-genital chancres would ‘be found upon investigation to be innocent in origin’ produced notable changes in medical practice.^104^ Standards for determining whether a given infection had been contracted ‘in an innocent or guilty manner’ often varied from physician to physician.^105^ Some automatically defaulted to *syphilis insontium* whenever it proved impossible to determine the exact origin of a patient’s infection.^106^ Others awarded the diagnosis to any patient whose genitals were free of sores and who denied that their other symptoms had a venereal origin.^107^ The most generous diagnosed *syphilis insontium* whenever their queries were answered with claims of sexual innocence.^108^

As this suggests, *syphilis insontium* did have some destigmatising effects.^109^ Overall, however, doctors were quite selective in their application of this concept. In most cases, their diagnostic determinations were guided by considerations of morality and class. For his part, Bulkley insisted that *syphilis insontium* was most often encountered ‘in good circles of society’; its chief victims included the ‘gentleman with a grown-up family’, the ‘man of high moral character’, and other ‘persons of unblemished purity’.^110^ Similarly reserving diagnoses of *syphilis insontium* for those believed to be morally virtuous, Bulkley’s allies aimed to convince readers that ‘many *respectable people* acquire syphilis innocently’.^111^ Their case reports were littered with remarks such as ‘habits of life good’, ‘morality was above suspicion’, and ‘morals reasonably good’.^112^

In keeping with Bulkley’s view that those syphilitics found ‘perfectly innocent of sexual transgressions’ tended to overlap with ‘those in the highest walks of life’, doctors generally inferred morality from socioeconomic status.^113^ A typical case involved a thirty-three-year-old ‘lady of refinement’ who visited Bulkley’s office with a curious eruption upon the soles of her feet and the palms of her hands.^114^ Upon questioning her, Bulkley learned that this woman had earlier suffered from ‘a stinging pain in the right tonsil’.^115^ Although her local doctor had pronounced this tonsillitis, Bulkley rejected this and ultimately diagnosed her with extra-genital syphilis. ‘No cause of infection could be learned’, he conceded, before concluding that the most likely culprit was ‘a public drinking cup, as she travelled much by cars, and drank often at the water cooler’.^116^

Assumptions of innocence were less common in cases involving individuals from more humble backgrounds. When treating a fifty-one-year-old Californian miner with a chancre of the lower lip, for example, Bulkley was unable to discover any ‘definite facts…in regard to the mode of infection’. Declining to render an opinion, Bulkley simply noted that this man ‘lived in a mining district, had smoked various pipes lying around in an assayer’s office…and had kissed girls of doubtful character’.^117^ His relatively apathetic approach to cases such as these reflected Bulkley’s broader views about people at the ‘lowest levels of society’ – whom he characterised as ignorant, careless, reckless, indifferent reservoirs of infection.^118^ Instead of considering the extent to which patients in the ‘poorer classes’ might themselves be suffering from non-venereal forms of syphilis, Bulkley presented these individuals as a danger to the ‘innocent’.^119^ His colleagues agreed that those responsible for spreading non-venereal forms of syphilis belonged to the ‘degraded and vicious classes’, including the ‘lady friend of questionable character’ and the ‘drunken loafer’.^120^

Those with a recent history of sexual contact were also generally thought to be ineligible for diagnoses of *syphilis insontium.* Though acknowledging that this could be acquired through ‘lawful coitus’, Bulkley’s definition of *syphilis insontium* as the ‘innocent acquirement of syphilis aside from coitus’ placed these cases outside the concept’s diagnostic boundaries.^121^ His colleagues replicated this approach, contending that ‘syphilis not due to sexual relations is called *syphilis of the innocent*’ and assuming that ‘the majority of cases of innocent syphilis must be extragenital’.^122^ The attempt to destigmatise syphilis by highlighting the cases of individuals ‘who certainly had not been exposed through sexual congress’ presumed that anyone who contracted the disease through sexual contact was guilty.^123^ The default assumption was to regard all genital sores with ‘suspicion’, and to hold these patients as ‘guilty…until they are proved innocent’.^124^

It was for this reason that prostitutes could not be deemed innocent even when they acquired syphilis non-sexually.^125^ When confronted by a nineteen-year old woman with a sore on her lower lip, Bulkley concluded that ‘as she appeared to be of a loose character’, it was ‘not difficult’ to understand the source of her infection – even in spite of the fact that no definitive history of this could be obtained.^126^ Similar refusals awaited those found to have indulged in sexual improprieties. In a case involving a thirty-year-old clerk with chancre of the upper lip, Bulkley refused to consider a diagnosis of *syphilis insontium* on account of the fact that the man was ‘dissipated, and subsequently returned with gonorrhea’; as such, it was ‘easy to understand that the lip infection took place in promiscuous intercourse’.^127^ Other physicians agreed with Bulkley’s idea that some extra-genital chancres occurred in ‘persons of loose habits, and were undoubtedly acquired in connection with venereal excitement’.^128^ In support of this, a doctor reported the following case:

A gentleman had presented himself with a chancre on his left shoulder: he could not understand how he had got it, admitting, however, that he was no Joseph. By helping his memory, he remembered that a couple of months previously he had carried a naked woman on his shoulders, and that he himself on this occasion had been as ‘when he came into this world.’ The conundrum was solved: he had, of course, had an acne on his shoulder and the woman probably papules on her genitalia. Such a case we cannot well call syphilis insontium.^129^

As all of this suggests, the stigma reduction goals advanced by Bulkley and other proponents of *syphilis insontium* were quite limited. Instead of aiming to destigmatise syphilis in the broadest possible sense, their pleas for tolerance and sympathy were meant to apply only to a narrow range of cases. Wedded to the idea that ‘the innocent too often suffer for the guilt of others’, Bulkley and his allies frequently linked their diagnoses of *syphilis insontium* to the immoral sexual behaviour of others.^130^ In insisting that innocents suffering from syphilis had been ‘sinned against’, Bulkley’s allies made morality matter in a way that directly undercut their explicit goals. An exclusionary term, *syphilis insontium* served primarily to shield those said to be of ‘good moral character’ and who could be given the benefit of the doubt; relatively few met these benchmarks.^131^ As a result, instead of undercutting the ‘wages of sin’ discourse, the promotion of *syphilis insontium* actually upheld that stigmatising mentality.

## ‘Ethics have no place in pathology’

With the passage of time, the counterproductive nature of Bulkley’s discourse became clear. In fact, several of his contemporaries predicted that his destigmatisation strategy would only solidify moral views of syphilis. Particularly pervasive was this criticism in the British Isles, where a noted obstetrician dubbed *syphilis insontium* ‘one of the most pathetic and tragic of all of the phrases in the terminology of medicine’.^132^ Though some British writers witnessed cases of *syphilis insontium* ‘capable of the most rigid proof’, most despaired of the concept, believing that it amplified the self-same stigmas it was purportedly designed to reduce. Arguing that *syphilis insontium* was a ‘thoroughly unscientific expression’, one doctor chastised Bulkley for his moralising, noting that ‘ethics [should] have no place in pathology’.^133^ Echoing this view, a 1914 article in *The Quarterly Review* judged the label *syphilis insontium* ‘unfortunate’, as it ‘impl[ied] that the disease in other instances was a syphilis of the guilty, a distinction which is quite outside the purview of medicine’.^134^ In his 1919 book *The Venereal Problem*, the English doctor Edmund Burke similarly concluded that ‘the term ‘innocent syphilis’ is a bad one’, as ‘there is no such thing as innocent or guilty syphilis’.^135^

Interestingly, some of Bulkley’s supporters agreed with this, acknowledging that *syphilis insontium* was an ‘ethical’ rather than a ‘medical’ concept, and that as such, it was ‘inadmissible’ on scientific grounds.^136^ Going beyond this concession, in an article that extended Bulkley’s ideas to gonorrhoea, the leading social hygienist Prince Morrow confessed that the term ‘innocent’ carried with it a ‘certain stigma’, as it signalled a belief that the malady in question was, ‘in some sort…a reproach to the bearer’, furnishing ‘presumptive proof…of immorality’.^137^

With the passage of time, criticisms of *syphilis insontium* became increasingly prominent in American medical literature as well. Though continuing to employ the term, physicians began to note its deficiencies, as did one doctor in 1910 when he explained that ‘by this use of the word syphilis innocently acquired, I do not wish to cast any reflections whatever upon those who have acquired it in any other manner’. ‘It is absolutely out of our province, as medical men’, he continued, ‘to consider for a moment the moral aspects of how a disease is contracted’.^138^ Agreeing with this, an Iowa doctor declared that no progress against stigma would be made ‘until we can look upon the victim of venereal disease, *however acquired*, as the most unfortunate of mortals’.^139^ ‘Perhaps he has not innocently acquired this disease’, a colleague concurred, ‘but in any case, physicians should give him due consideration’.^140^ Believing that ‘the innocent and guilty are so mingled that only the All-Seeing and All-Knowing can absolutely separate them’, in 1915, a Boston-based venereologist declared it the goal of medicine ‘to seek and save rather than to judge in a cause of which our knowledge is at best very imperfect’.^141^ One year later, a thoroughgoing repudiation of Bulkley’s ideas appeared in the pages of *Social Hygiene*:

We must have a new public attitude toward infectious diseases…In the past we have judged people and consigned them to punishment because of the infection of venereal disease as if the punishment in the disease itself was insufficient. *Who are we that we should judge the victim of venereal disease, innocent or guilty?* How do we know why a man or woman acquires the disease which in one social state is a vice, in another a woeful accident or marriage. And if we are to constitute ourselves as judges, how many among us can say, ‘I never had an infectious disease; I never sinned’, or ‘I was never tempted.’ And if I never sinned, was it only accident and not self-restraint that I did not? Therefore, if we cannot condone the iniquity let us at least treat the patient as a man or woman and not deny him treatment and abuse him with the cruelty of a beast.’^142^

The recognition that Bulkley’s approach to stigma reduction could actually amplify syphilis’ association with guilt and immorality emerged almost as rapidly as did the initial embrace of his ideas. In 1910, for example, a New York doctor wrote of how it was ‘not so very long ago that an elaborate treatise on syphilis of the innocent was published here; as if all syphilis was not syphilis of the innocent – or as if we, as medical men, could say of any case of luetic infection, that it was deserved’.^143^ Given the swiftness with which the medical profession turned against Bulkey’s ideas in the first few years of the twentieth century, it may be tempting to pin this sudden about-face on contemporaneous developments in medical knowledge and practice – for example, the invention of the Wassermann test in 1905. Did the emergence and rapid adoption of this new diagnostic technology contribute to the growing criticism of Bulkley’s ideas, and to the emerging consensus that ‘there is no such thing as innocent or guilty syphilis’?

A full exploration of this question remains outside of the scope of the present study. Nevertheless, there are reasons to doubt the existence of a causal relationship between venereological advances and the profession’s thoroughgoing re-evaluation of Bulkey’s ideas. For one, although the Wassermann test gave doctors an additional means for diagnosing syphilis, it offered no assistance in helping them identify means of disease transmission. When deciding whether or not to award a diagnosis of *syphilis insontium* to their patients, Bulkley and his colleagues did so on the basis of clinical examinations and case histories, not laboratory findings. This remained as true after the introduction of the Wassermann test as it did before.^144^ Rather than prompting a reconsideration of Bulkley’s theories, doctors often employed this new diagnostic procedure in the hopes that it would help those believed to be suffering from an innocently acquired case of syphilis.^145^

As this suggests, the turn against Bulkley’s approach to stigma reduction did not spell the end of the history of *syphilis insontium* as a disease concept. If anything, the passage of time yielded a significant expansion of the category of ‘innocently acquired syphilis’. As an indication of how the diagnostic criteria surrounding this concept loosened, an article published in 1922 proclaimed that ‘if the source and mode of infection be within the realms of possibility and probability, it seems just to accept the cause as related to us by the patient’.^146^ With this, new possibilities for destigmatisation emerged – even among individuals whose sexual practices were generally deemed immoral. In 1921, for example, a doctor published a case involving a twenty-eight-year-old man who sought medical assistance for a sore on his gums. Said to display ‘all the earmarks of homosexuality’, the man ‘frankly admitted perversion of the sexual act’ – specifically, oral sex, which had been practiced at ‘regular intervals for a number of years’.^147^ Examination revealed no other signs of syphilis, but instead of concluding that his oral lesion had resulted from ‘perversion’, the attending physician considered it equally likely that this had resulted from a recent trip to the dentist in which the man’s left canine tooth had been extracted. Believing that the ‘chief source’ of such infections was ‘not through venery, but is accidental’, this physician was willing to confer a diagnosis of *syphilis insontium* even in light of evidence to the contrary.^148^

Further evidence of the staying power of *syphilis insontium* can be seen in the publication of a 1922 volume that bore the same name as Bulkley’s 1894 text.^149^ Following their predecessor, its authors argued that there were ‘many instances of extragenital infection’ via syphilis.^150^ But whereas Bulkley’s writings focused primarily on non-venereal modes of transmission, this text observed that ‘sexual intercourse is the most frequent method by which the disease’ is spread.^151^ And when speaking of ‘syphilis acquired innocently’, this study included sexual modes of transmission – most notably, ‘syphilis contracted by either husband or wife in the marriage state’.^152^

These changes presaged developments of the next two decades. In her analysis of the federal government’s response to syphilis during the Great Depression and the Second World War, Erin Wuebker argues that the efforts of influential public health figures such as Surgeon General Thomas Parran succeeded in ‘opening up unforeseen opportunities for care and destigmatised meanings of VD’.^153^ Though VD discourses of the 1930s and 1940s similarly focused on the idea of innocent infections, they generally centred on the family, and succeeded in ‘avoiding much discussion of how the disease was originally acquired or blaming particular partners’.^154^ By deemphasising questions of how syphilis was being transmitted, this version of Bulkley’s ideas helped spur bold, decisive actions to reduce the disease’s prevalence. The origins of this new, more productive approach to ‘syphilis of the innocent’ can be traced back to the critique of Bulkley’s ideas that emerged in the first decade of the twentieth century.

## Conclusion

The evolution of *syphilis insontium* through the late nineteenth and early twentieth centuries speaks to the constancy and inescapability of metaphors in human understandings of disease. In her writings, Susan Sontag warned against metaphorical thinking, arguing that metaphors not only impede objective, accurate understandings of illness but also isolate and stigmatise the sick in ways that harm well-being and hamper recovery. Critics of Sontag’s efforts to make medical thought immune to metaphors have pointed out how her positivist approach betrays a fundamental discomfort with the socially constructed nature of disease – and an inability to recognise that ‘stopping metaphors is like ceasing to eat or breathe’.^155^ Instead of eradicating metaphors through a ‘retreat into the radical materialism of biomedicine’, these critics contend, the goal should be to treat metaphors as a means of empowerment – that is, as a way to promote approaches to illness that ‘decrease suffering rather than add to it’.^156^

The first chapter of the history of *syphilis insontium* highlights some of the ways that disease metaphors can exacerbate existing stigmas and actively harm individuals. As has been seen, Lucius Bulkley’s campaign to sever syphilis’ associations with sexual immorality offers a historical illustration of Sontag’s argument about how ideas of innocence can reify and deepen discourses of shame, blame, and guilt. However, as the critique and subsequent evolution of *syphilis insontium* indicates, these associations between innocent victims and guilty perpetrators are not inherent to the metaphor. By the 1920s, a new understanding of *syphilis insontium* was beginning to emerge, and in the 1930s, this developed into a much more productive stigma reduction campaign. Metaphors of innocence, then, need not necessarily direct attention to the moral failings of others. When deployed properly, these metaphors can be used to underscore the idea that all those infected with a given disease are unfortunate and fully worthy of the utmost compassion.

As stigma remains an ever-present reality in the contemporary world, much is to be gained from studying past destigmatisation efforts. Though the evidence presented here pertains solely to the problematic features of these efforts, future research on this subject may yield more evidence of wholly successful strategies. Regardless, acquiring a better knowledge of what has worked and what has not is essential to formulating evidence-based approaches to destigmatisation. Without this, our efforts, like those of Lucius Bulkley and his allies, will only succeed in furthering the prejudices and negative perceptions they endeavour to undercut. And that is something we simply cannot afford.

## Footnotes

1

L. Duncan Bulkley , ‘Syphilis as a Non-Venereal Disease, With a Plea for the Legal Control of Syphilis’, Journal of the American Medical Association, 36, 14 (1901), 933 .

2
*Ibid.*, 934.

3
*Ibid.*, 933.

4
*Ibid.*, 933.

5
*Ibid.*, 933.

6
*Ibid.*, 938, 934.

7
*Ibid.*, 934.

8
*Ibid.*, 935, 938.

9
*Ibid.*, 934, 936.

10
*Ibid.*, 937-8.

11
*Ibid.*, 937.

12 Erin Wuebker, ‘Taking the Venereal out of Venereal Disease: The Public Health Campaign Against Syphilis, 1934-1945’, (City University of New York, 2015); William Anderson , ‘“The Next Great Plague to Go”: How the U.S. Surgeon General Used Public Relations to Fight Venereal Disease during the Great Depression’, Journalism History 44:2 (2018), 63–9.

13

Henk van den Belt , Spirochaetes, Serology, and Salvarsan: Ludwik Fleck and the Construction of Medical Knowledge about Syphilis (Grafisch Bedrijf Ponsen & Looijen B.V., 1997), 71–3.

14

Susan Sontag , AIDS and its Metaphors (Farrar, Straus, and Giroux, 1990), 39 .

15 Philip J. Fleming, ‘Shadow Over New Zealand’: The Response to Venereal Disease in New Zealand, 1910-1945’ (Massey University, 1989), 134.

16

Monika Pietrzak-Franger , ‘Medical Mappings of Syphilis in the Late Nineteenth Century’, in Kari Nixon and Lorenzo Servitje (eds.), Syphilis and Subjectivity: From the Victorians to the Present (New York: Palgrave Macmillan, 2018), 30 .

17

Elisabet Björklund , ‘Sins of the fathers?: Syphilis, HIV/AIDS, and innocent women and children’, in A Visual History of HIV/AIDS (Routledge, 2018), 178 .

18 Wuebker, *op. cit.* (note 12).

19 For examples of this, see Barbara Clow , ‘Who’s afraid of Susan Sontag? Or, the myths and metaphors of cancer reconsidered’, Social History of Medicine 14, 2 (2001), 293–312 11697354 10.1093/shm/14.2.293.

20 Bulkley, *op. cit.* (note 1), 933.

21

John L. Coffin , ‘The Extra-Genital Primary Lesion of Syphilis’, The New-England Medical Gazette, 31, 11 (1896), 512 .

22 Bulkley, *op. cit.*, (note 1), 933.

23

L.D. Bulkley , ‘Two Cases of Chancre of the Lip Probably Acquired Through Cigars’, Transactions of the American Dermatological Association, 3 (1880), 47 .

24

Abraham L. Wolbarst , ‘The Venereal Diseases: A Menace to the National Welfare’, Medical Review, 62 (1910), 327–80; L. Duncan Bulkley , Syphilis in the Innocent (Bailey and Forchild, 1894), 112 .

25 Oriel, *op. cit.* (note 48). See also Karen J. Taylor , ‘Venereal Disease in Nineteenth-Century Children’, The Journal of Psychohistory, 12, 4 (1985), 431–6311620715 .

26

L. Duncan Bulkley , ‘Plain Truths about Syphilis’, Indiana Medical Journal 26:3 (1907), 103 . For more, see G. Shearman Peterkin , ‘A System of Venereal Prophylaxis That is Producing Results’, American Medicine, 10 (1906), 328 ; C. Cunningham , ‘The Importance of Venereal Disease’, The New England Journal of Medicine, 168, 3 (1913), 77–8; ‘Therapeutic Nuggets’, *The American Journal of Clinical Medicine*, 15 (1908), 1296; Allan McLane Hamilton , A System of Legal Medicine, Volume 2 (New York: E.B. Treat, 1895), 489 .

27

Abraham L. Wolbarst , ‘The Venereal Diseases: A Menace to the National Welfare’, Medical Review, 62 (1910), 373 .

28

Walter D. Bierbach , ‘Venereal Disease and Prostitution’, Boston Medical and Surgical Journal, 172 (1915), 205 ; ‘Report of the Red Plague Committee’, *Transactions of the Commonwealth Club of California*, 8 (1913), 340.

29 For more on these developments, see J.D. Oriel , Scars of Venus: A History of Venereology (Springer-Verlag, 1994).

30 See, in particular, Allan Brandt , No Magic Bullet: A Social History of Venereal Disease in the United States since 1880 (Oxford University Press, 1987).

31 For more on this, see John Parascandola , Sex, Sin, and Science: A History of Syphilis in America (Westport: Praeger, 2008), 39–45 .

32 Wolbarst, *op. cit.* (note 26), 327.

33 For more on this, see Henry C. McHatton , The Venereal Peril: Address to the Young Men of the Young Men’s Christian Association (Macon: Smith & Watson, 1907). For more on the social hygiene movement, see Robin E. Jensen , Dirty Words: The Rhetoric of Public Sex Education, 1870-1924 (Urbana: University of Illinois Press, 2010), 17–23 .

34 Brandt, *op. cit.* (note 29), 182.

35 For relevant examples, see Suzanne Poirier , Chicago’s War on Syphilis, 1937-40: The Times, the Trib, and the Clap Doctor (Urbana: University of Illinois Press, 1995); Nancy K. Bristow , Making Men Moral: Social Engineering during the Great War (New York: New York University Press, 1996); Marilyn Hegarty , Victory Girls, Khaki-Wackies, and Patriotutes: The Regulation of Female Sexuality during World War Two (New York: New York University Press, 2008); Elliott Bowen , In Search of Sexual Health: Diagnosing and Treating Syphilis in Hot Springs, Arkansas, 1890-1940 (Baltimore: Johns Hopkins University Press, 2020).

36

Theodore Schroeder , Freedom of the Press and “Obscene” Literature: Three Essays (New York: The Free Speech League, 1906), 25 ; Frank Wieland , ‘Treatment of Gonorrhea’, The Medical Forum, 1, 5 (1904), 198 .

37

Ronald Bayer , ‘Stigma and the Ethics of Public Health: Not Can We But Should We’, Social Science & Medicine 67 (2008), 463–7218502551 10.1016/j.socscimed.2008.03.017.

38

L.H. Ban Buskirk , ‘The Laboratory and Venereal Diseases’, Ohio Public Health Journal 9:7 (1918), 305 ; Schroeder, *op. cit.* (note 35), 25.

39 Brandt, *op. cit.* (note 29), 12.

40

Roger Davidson , Dangerous Liaisons: A Social History of Venereal Disease in Twentieth-Century Scotland (Rodopoi, 2000), 54–511027064.

41 ‘Editorial: The Moral Etiology of Syphilis’, *Journal of Cutaneous Diseases Including Syphilis* 21:2 (1903), 57.

42

William Driscoll , ‘The Metaphor of Syphilis in Grand’s *Heavenly Twins*
’, Nineteenth-Century Gender Studies 5:1 (2009), 3 .

43
*Ibid.*, 14.

44

Herman Goodman , Notable Contributions to the Knowledge of Dermatology (New York: Medical Lay Press, 1953), 278 .

45

Herman Goodman , ‘Lucius Duncan Bulkley’, Medical Life, 35 (1928), 400 .

46 ‘L. Duncan Bulkley’, *Columbia Alumni News*, 20 (1928), 13.

47

James J. Walsh , History of Medicine in New York (New York: National Americana Society), 61 .

48

L.D. Bulkley , ‘Seven Cases of Palmar Syphilis’, New York Medical Journal, 26, 4 (1877), 410 .

49

L.D. Bulkley , ‘On Unusual Methods of Acquiring Syphilis: With Reports of Cases’, Medical News, 54, 9 (1889), 232–4, 266.

50 L. Duncan Bulkley, ‘Non-Venereal Syphilis’, *Transactions of the Medical Society of the State of New York For the Year 1886* (1886), 402; Bulkley, *op. cit.* (note 20), 47; Bulkley, *op. cit.* (note 26); 234.

51 L. Duncan Bulkley, ‘The Malignity of Syphilis, With an Analysis of Four Hundred and Fifty Cases of the Disease’, *Transactions of the Medical Society of the State of New York For the Year 1882* (1882), 248.

52 Bulkley, ‘Should Education in Sexual Matters Be Given to Young Men of the Working Classes?’, *Interstate Medical Journal*, 13, 3 (1906), 302; Bulkley, *op. cit.* (note 27), 402; Bulkley, *op. cit.* (note 20), 47; Bulkley, *op. cit.* (note 26), 234.

53 For earlier discussions of this, see S.W. Butler and D.G. Brinton , Half-Yearly Compendium of Medical Science: A Synopsis of the American and Foreign Literature of Medicine, Surgery, and the Collateral Sciences, for Six Months, Part II (Philadelphia: S.W. Butler 1868), 332 .

54

M.H. Henry , ‘Clinical Contributions: Three Cases of Indurations of the Os and Cervix Uteri, The Result of Syphilis; Two Cases of Syphilitic Insanity; Four Cases of Anomalous Localities of Chancres, Extra-Genital, With Remarks’, American Journal of Syphilology and Dermatology, 6, 1 (1874), 356 .

55

R.W. Taylor , ‘Syphilis and Venereal Diseases: General Questions in Syphilis, Therapeusis, Etc.’, Archives of Dermatology, 2 (1875), 359 ; ‘Letter to Dr. E.S Frazer From His Son, S.H. Frazer, In Paris’, *Saint Louis Medical and Surgical Journal*, 4, 6 (1867), 543, 546.

56

Gottlieb Heinrich Georg Jahr , The Venereal Diseases: Their Pathological Nature, Correct Diagnosis, and Homeopathic Treatment (New York: William Radde, 1868), 99 .

57 ‘Extra-Genital Chancres’, *St. Louis Medical and Surgical Journal*, 48, 5 (1885), 401.

58 Bulkley, *op. cit.* (note 26), 234.

59
*Ibid.*, 234.

60
*Ibid.*, 234.

61
*Ibid.*, 234.

62 For more on this, see ‘Survival of T. pallidum outside the body: I. General observations’, *Bulletin of the World Health Organisation*, 35 Supplement (1966), 78-85.

63 For more on the discovery of asymptomatic carriers, see Judith Walzer Leavitt , Typhoid Mary: Captive to the Public’s Health (Boston: Beacon Press, 1996).

64

Peter C. Baldwin , ‘Dangers that Lurk in a Kiss: Quarantining the American Mouth, 1890-1920’, Journal of Social History 55, 3 (2022), 647–67.

65

Lucius Duncan Bulkley , Syphilis of the Innocent (New York: Bailey and Fairchild, 1894), 23 ; Bulkley, *op. cit.* (note 26), 234; Bulkley, *op. cit.* (note 27), 400.

66

L. Duncan Bulkley , ‘Syphilis as a Non-Venereal Disease’, Journal of the American Medical Association, 11, 25 (1888), 872 .

67 Bulkley, *op. cit.* (note 27), 402.

68 L. Duncan Bulkley, ‘Clinical Notes on Chancre of the Tonsil; With Analyses of Fourteen Cases’, *Transactions of the Medical Society of the State of New York For the Year 1893* (1893), 370.

69 L. Duncan Bulkley, ‘Manifestations of Syphilis in the Mouth’, *Transactions of the New York Odontological Society* (1898), 11-26.

70 Bulkley, *op. cit.* (note 26), 266.

71 Pietrzak-Franger, *op. cit.* (note 16), 21, 26.

72
*Ibid.*, 13.

73

L. Duncan Bulkley , ‘Some of the Difficulties in the Diagnosis and Treatment of Syphilis’, Virginia Medical Monthly, 9, 7 (1882), 391 ; Bulkley, *op. cit.* (note 20), 48.

74 Bulkley, *op. cit.* (note 45), 363; Bulkley, *op. cit.* (note 26), 232.

75 For historical analyses of other early twentieth-century disease destigmatisation efforts, see Elizabeth Toon , ‘“Cancer as the General Population Knows It”: Knowledge, Gear, and Lay Education in 1950s Britain,’ Bulletin of the History of Medicine 81 (2007), 116–3817369665 10.1353/bhm.2007.0013PMC2635844; Vicki Long , Destigmatising Mental Illness? Professional Politics and Public Education in Britain, 1870-1970 (Manchester, UK: Manchester University Press, 2015).

76 For a contemporary example of this, see C. Sonnex , ‘Destigmatizing Genitourinary Medicine and Sexually Transmitted Infections in the UK,’ International Journal of STD & AIDS 20:11 (2009), 748–5119854887 10.1258/ijsa.2009.009312.

77 For an introduction to the sociological literature on destigmatisation, see Matthew Clair , Caitlin Daniel , and Michèle Lamont , ‘Destigmatization and Health: Cultural Constructions and the Long-Term Reduction of Stigma’, Social Science & Medicine 165 (2016): 223–232 27020492 10.1016/j.socscimed.2016.03.021PMC5758051. For more on normalisation as a stigma reduction practice, see Ilse J. Volinn , ‘Health Professionals as Stigmatizers and Destigmatizers of Diseases: Alcoholism and Leprosy as Examples.’ Social Science & Medicine 17, no. 7 (1983): 385–393 6867781 10.1016/0277-9536(83)90341-6; Liora Navon , ‘Beyond Constructionism and Pessimism: Theoretical Implications of Leprosy Destigmatisation Campaigns in Thailand’, Sociology of Health & Illness 18, no. 2 (1996): 258–276 ; Liz Sayce , From Psychiatric Patient to Citizen: Overcoming Discrimination and Social Exclusion (New York: St. Martin’s Press, 2000).

78

L. Duncan Bulkley , ‘Unrecognized Syphilis in General Practice’, International Medical Magazine 10:12 (1901), 720 .

79 Bulkley, *op. cit.* (note 1), 937.

80
*Ibid.*, 937.

81 Bulkey, *op. cit.* (note 78), 719.

82 Bulkley, *op. cit.* (note 73), 390; Bulkley, *op. cit.* (note 1), 936.

83 Bulkley, *op. cit.* (note 1), 937.

84

L. Duncan Bulkley , ‘Syphilis Insontium: A Plea for the Restriction of Syphilis, and a Suggestion for the Prevention of Its Spread’, Journal of the American Medical Association 21:20 (1893), 734 ; L. Duncan Bulkley , ‘Non-Venereal Syphilis’, Transactions of the Medical Society of the State of New York (1886), 394 .

85 L. Duncan Bulkley, *op. cit.* (note 27), 396; L. Duncan Bulkley , ‘Some Plain Truths About Syphilis’, Medical Record, 72, 6 (1907), 214 .

86 Bulkley, *op. cit.* (note 45), 371-2.

87 Bulkley, *op. cit.* (note 27), 394; Bulkley, *op. cit.* (note 43), 870.

88

L. Duncan Bulkley , ‘Syphilis Occurring in Connection with Other Diseases of the Skin’, Transactions of the Medical Society of the State of New York (1887), 449 .

89 Bulkley, *op. cit.* (note 29), 303.

90 Ibid., 304.

91 Bulkley, *op. cit.* (note 1), 938.

92 For evidence of the impact of Bulkley’s writings, see Walter F. Chappell , ‘Two Cases of Chancre of the Tonsil’, Medical Record, 45, 4 (1894), 104 ; I.N. Bloom , ‘Some Extra-Genital and Other Chancres’, The Medical Age, 16, 18 (1898) 554–9; John Edwin Rhodes , ‘Chancre of Tonsil, With Report of Thirty-Five Cases’, Transactions of the Twenty-Third Annual Meeting of the American Laryngological Association (1901), 27–38 ; G.A. Aschman , ‘Primary Syphilis of the Faucial Tonsil Treatment of Syphilis by Inunctions’, Transactions of the Medical Society of the State of West Virginia (1903), 195–202 ; Nelson D. Brayton , ‘Syphilis,—A Non-Venereal Disease’, The Medical and Surgical Monitor, 7 (1904), 301–8; ‘Around the Table’, *The Medical Standard*, 28 (1905), 681; A.R. Thrasher, ‘Primary Syphilis of Lips and Tonsils’, *Transactions of the Thirteenth Annual Meeting of the American Laryngological, Rhinological, and Otological Society* (1907), 462-6. For criticism of Bulkley, see Bernard Wolff , ‘Extra-Genital Syphilis in Some of Its Sociologic Relations’, Virginia Medical Semi-Monthly, 3, 13 (1898), 367–9.

93

Alfred E. Diehl , ‘Five Cases of Extra-Genital Chancre’, Buffalo Medical Journal, 36 (no. 9, April 1897), 671 PMC866049836884544.

94 For evidence of this, see Burnside Foster , ‘Report of Cases of Extragenital Chancre’, Northwestern Lancet, 17 (1897), 395 ; Francis Denison Patterson , ‘Extra-Genital Chancre—Infection from a Barber’, Therapeutic Gazette, 21, 11 (1905), 740–1.

95

M.H. Henry , ‘Bumstead on Venereal Diseases’, American Journal of Syphilography and Dermatology, 2 (1871) 63, 64.

96

S.G. Dabney , ‘Chancre of the Tonsil’, American Practitioner and News, 151, 6 (1901), 222 . Emphasis added. For the second quote, see T.C. Evans , ‘Chancre of the Tonsil and Tongue, with Report of Four Cases’, Transactions of the Second Annual Meeting of the American Laryngological, Rhinological, and Otological Society (1896), 40 .

97 ‘Extra-Genital Chancres’, *The Medical World*, 109, 1 (1901), 5.

98 Bloom, *op. cit.* (note 70), 555.

99

William S. Gottheil , ‘The Diagnosis of the Extra-Genital Chancre’, The Archives of Diagnosis, 4, 2 (1911), 155 .

100 Coffin, *op. cit.* (note 12), 512.

101

Ralph Williams , ‘Syphilis Extra-Genital Chancres’, The Southern California Practitioner, 20, 12 (1905), 546 .

102 Bulkley, *op. cit.* (note 63), 213.

103

Follen Cabot , ‘The Management of Syphilis’, American Journal of Dermatology and Genito-Urinary Diseases, 2, 4 (1898), 108 ; Divya Cherian , ‘Stolen Skin and Children Thrown: Governing Sex and Abortion in Early Modern South Asia’, Modern Asian Studies, 55 (5), 1461–1509 .

104 Cabot, *op. cit.* (note 80), 108.

105

Rev. Stanislaus , The Casuist: A Collection of Cases in Moral and Pastoral Theology, Volume 4 (New York: Joseph F. Wagner 1912), 299 .

106

Robert W. Taylor , ‘Does Syphilis in Some Cases Spontaneously Abort in the Primary Stage?’ The American Journal of Surgery, 20, 11 (1906), 322 .

107

J.L. Belknap , ‘Syphilis Insontium’, *Report of the Surgeon-General, U.S. Navy* (Washington: Government Printing Office, 1905), 193 .

108

E.P. Stone , ‘Chancre of the Tonsil’, The Medical Age, 15, 3 (1897), 65–75 .

109 As an example of this, see Rhodes, *op. cit.* (note 70), 30-1. For a similar case, see Arthur G. Root, ‘Primary Syphilitic Lesion of the Tonsil’, *Transactions of the Third Annual Meeting of the American Laryngological, Rhinological, and Otological Society* (1898), 74.

110 Bulkley, *op. cit.* (note 63), 213; Bulkley, *op. cit.* (note 26), 234**;** L.D. Bulkley, ‘Notes on Recent Cases of Extra-Genital Syphilitic Infection’, *Journal of the American Medical Association*, 38, 2 (1902), 100; Bulkley, *op. cit.* (note 61), 391.

111

Walter Brooks Brouner , ‘Innocently Acquired Syphilis’, New York Medical Journal, 92, 7 (1910), 319 .

112

Charles F. Kuhn , ‘Some Obscure Cases Difficult to Diagnose’, American Journal of Dermatology and Genito-Urinary Diseases, 15, 6 (1911), 312 ; Henry A. Robbins , ‘Non-Venereal Syphilis’, Virginia Medical Monthly, 21, 7 (1894), 583 ; Williams, *op. cit.* (note 79), 547.

113 Bulkley, *op. cit.* (note 66), 448.

114 Bulkley, *op. cit.* (note 42), 72.

115
*Ibid.*, 72.

116
*Ibid.*, 72.

117 Bulkley, *op. cit.* (note 42), 61.

118 Bulkley, *op. cit.* (note 29), 302.

119
*Ibid.*, 303.

120

Henry A. Robbins , ‘Syphilis of the Innocent’, Maryland Medical Journal, 36, 15 (1897), 262 ; Hicks C. Fenton , ‘Primary Tonsillar Syphilis, With Report of a Case’, Medical Sentinel, 2, 2 (1899), 251 ; William F. Barclay , ‘The Dangers of Syphilitic Infection from Non-Sexual Causation’, The Chicago Clinic, 11, 9 (1898), 218 .

121 Bulkley, *op. cit.* (note 28), 248; Bulkley, *op. cit.* (note 96).

122

John Chalmers da Costa , Modern Surgery: General and Operative (Philadelphia: W.B. Saunders Company 1910), 313 ; Thomas Paul , ‘Should Venereal Infections Be Classed and Reported as Contagious Diseases?’ The Medical Herald, 30, 10 (1911), 439 . The speaker in this instance was Dr W.L. Kenney.

123 Bulkley, *op. cit.* (note 26), 266.

124

E. Wood Ruggles , ‘The Management of Genital Sores’, New York Medical Journal and Philadelphia Medical Journal: A Weekly Review of Medicine, 80, 8 (1904), 338 .

125 For an example of this, see Joseph Grindon , ‘Extra-Genital Chancre’, Medical Review, 35, 20 (1897), 349 .

126
*Ibid.*, 41-2.

127 Bulkley, *op. cit.* (note 42), 53.

128 Bulkley, *op. cit.* (note 27), 396.

129

Burnside Foster , ‘On the Extragenital Chancre, Its Frequency and Its Significance for the Course of Syphilis’, Saint Paul Medical Journal, 1, 8 (1899), 632 .

130

George Henry Fox , ‘Oral Chancre: Cases and Comments’, International Record of Medicine and General Practice, 31 (1880), 145 ; William Warren Townsend , ‘Venereal Disease in Vermont’, Vermont Medical Monthly, 16, 1 (1910), 66–75 .

131 Stout, *op. cit.* (note 119), 979.

132

John William Ballantyne , Expectant Motherhood: Its Supervision and Hygiene (London: Funk and Wagnalls Company, 1914), 134 .

133

Sir Darcy Power and James Keogh Murphy , A System of Syphilis, Vol. 2 (London: Frowde, 1908), 174 .

134 ‘Syphilis’, *The Quarterly Review*, 221-2 (1914), 151.

135

Edmund Tytler Burke , The Venereal Problem (London: H. Kimpton,1919), 40 .

136

Prince A. Morrow , ‘Gonorrhea Insontium, Especially in Relation to Marriage’, Bulletin of the American Academy of Medicine, 6, 7 (1903), 410 .

137
*Ibid.*, 410.

138

A.D. Mewborn , ‘What are the Facilities for Treatment Open to the Venereal Patient in the Dispensaries and Hospitals of New York?’ Transactions of the American Society of Sanitary and Moral Prophylaxis, 3 (1910), 76 .

139

H.B. Young , ‘Venereal Disease Innocently Acquired’, Iowa Medical Journal, 11, 11 (1905), 435 .

140

Edward L. Keyes , ‘Protection of the Innocent’, Journal of the American Medical Association, 47, 23 (1906), 1947 . The speaker here was one George Frank Butler, a doctor from Chicago who participated in the discussion following Keyes’ paper.

141

Abner Post , ‘Remarks at Neurosyphilis Conference’, Boston Medical and Surgical Journal, 173, 2 (1915), 868 .

142

George W. Goler , ‘The Municipality and the Venereal Disease Problem’, Social Hygiene, Volume 2 (1916), 60–1. Emphasis added.

143

William S. Gottheil , ‘Some Dermatological “Manias and Phobias”’, New York Medical Journal 91:13 (March 26, 1910), 628 .

144

Walter Brooks Brouner , ‘Innocently Acquired Syphilis’, New York Medical Journal 92 (1916), 317–20.

145 ‘Wassermann Reaction in Infants’, *Boston Medical and Surgical Journal* 166:13 (1912), 503-4.

146

John S. Lambie , ‘The Prevention of Extra-Genital Chancres in the Army, Based on a Study of Syphilitic Registers on File at the Army Medical Schools’, The Military Surgeon, 50, 3 (1922), 267 .

147

Joseph Victor Klauder , ‘Chancre of the Gum with Report of a Case’, Archives of Dermatology and Syphilology, 3, 5 (1921), 624 .

148 Ibid., 620.

149

Harry C. Solomon and Maida Herman Solomon , Syphilis of the Innocent: A Study of the Social Effects of Syphilis on the Family and the Community (Washington: United States Interdepartmental Social Hygiene Board, 1922).

150
*Ibid.*, 2.

151
*Ibid.*, 2.

152
*Ibid.*, 14.

153 Wuebker, *op. cit.* (note 12), 14.

154 Ibid., 76.

155

Allan M. Brandt , ‘AIDS and Metaphor: Toward the Social Meaning of Epidemic Disease’, Social Research 53:3 (1988), 413–3211650269; Martha Stoddard Holmes , ‘After Sontag: Reclaiming Metaphor’, Genre: Forms of Discourse and Culture 44:3 (2011), 264 .

156

Susan M. DiGiacomo , ‘Metaphor as Illness: Postmodern dilemmas in the representation of body, mind and disorder’, Medical Anthropology: Cross-Cultural Studies in Health and Illness 14:1 (1992), 117 10.1080/01459740.1992.99660681284168; Holmes, *op. cit.* (note 144), 269.

